# Population Structure of *Streptococcus pneumoniae* Causing Invasive Disease in Adults in Portugal before PCV13 Availability for Adults: 2008-2011

**DOI:** 10.1371/journal.pone.0153602

**Published:** 2016-05-11

**Authors:** Andreia N. Horácio, Catarina Silva-Costa, Jorge Diamantino-Miranda, Joana P. Lopes, Mario Ramirez, José Melo-Cristino, Portuguese Group for the Study of Streptococcal Infections

**Affiliations:** Instituto de Microbiologia, Instituto de Medicina Molecular, Faculdade de Medicina, Universidade de Lisboa, Lisbon, Portugal; Rockefeller University, UNITED STATES

## Abstract

Among the 1660 isolates recovered from invasive pneumococcal disease (IPD) in adults (> = 18 yrs) in 2008–2011, a random sample of ≥50% of each serotype (n = 871) was chosen for MLST analysis and evaluation for the presence and type of pilus islands (PIs). The genetic diversity was high with 206 different sequence types (STs) detected, but it varied significantly between serotypes. The different STs represented 80 clonal complexes (CCs) according to goeBURST with the six more frequent accounting for more than half (50.6%) of the isolates—CC156 (serotypes 14, 9V and 23F), CC191 (serotype 7F), CC180 (serotype 3), CC306 (serotype 1), CC62 (serotypes 8 and 11A) and CC230 (serotype 19A). Most of the isolates (n = 587, 67.3%) were related to 29 Pneumococcal Molecular Epidemiology Network recognized clones. The overall proportion of isolates positive for any of the PIs was small (31.9%) and declined gradually during the study period (26.6% in 2011), mostly due to the significant decline of serotype 1 which is associated with PI-2. The changes in serotypes that occurred in adult IPD after the introduction of the seven-valent pneumococcal conjugate vaccine (PCV7) for children were mostly due to the expansion of previously circulating clones, while capsular switching was infrequent and not related to vaccine use. The reduction of IPD caused by PCV7 serotypes in the years following PCV7 implementation did not result in a decline of antimicrobial resistance in part due to the selection of resistant genotypes among serotypes 14 and 19A.

## Introduction

The 7-valent conjugate vaccine (PCV7) was available for children through the private sector in Portugal from 2001 onwards until it was replaced in the beginning of 2010 by the 13-valent conjugate vaccine (PCV13). In 2012, PCV13 received approval for use also in adults > 50 years of age with an extension being made to all ages in 2013. Additionally, PCV13 entered the Portuguese National Immunization Program (NIP) in June 2015 for children born from January 2015 onwards. Two other vaccines, the 23-valent pneumococcal polysaccharide vaccine (PPV23) and the 10-valent conjugate vaccine (PCV10), have also been available in Portugal since 1996 and 2009, respectively, but with a low uptake [1].

Among the more than 90 different pneumococcal serotypes identified, only a few cause the majority of IPD. While for some serotypes the capsular polysaccharide is the dominant determinant of invasiveness, for others distinct genotypes show important differences in invasiveness [2]. Additionally, there are other features that are strongly associated with genotype independently of serotype, such as antimicrobial susceptibility and the presence and type of pilus islands [3,4]. With the availability of pneumococcal conjugate vaccines that efficiently target particular serotypes, important changes have been reported regarding not only serotype but also genotype distributions of pneumococci causing IPD [5–9]. Interestingly, while non-vaccine serotypes have emerged as a cause of IPD, in some cases distinct clones expressing the same serotype have risen in frequency in different geographic regions [10,11].

While numerous studies have addressed the serotype distribution of IPD, information regarding the clonal composition of pneumococcal populations has been scarcer. In a previous study we defined the clonal composition of pneumococci causing IPD in both children and adults in the pre-PCV7 period [11]. In a subsequent study we documented major changes in the potential coverage of PCV13 starting in 2009, due to decreases in prevalence of serotypes 1 and 5 [12]. In the present study we aimed to characterize the clonal composition of pneumococci causing adult IPD in Portugal between 2008 and 2011, a period characterized by extensive use of PCV7 and the adoption of PCV13 in children and prior to the use of PCV13 in adults.

## Materials and Methods

### Bacterial isolates

The isolates included in this study were recovered from adult patients (≥18 yrs) with invasive pneumococcal disease between 2008 and 2011 and were characterized in previous studies regarding serotype distribution and antimicrobial susceptibility [1,12]. A case of invasive disease was defined by the recovery of pneumococci from a normally sterile source, such as blood or cerebral spinal fluid (CSF). Serotypes were grouped into conjugate vaccine serotypes, i.e., those included in PCV13 (serotypes 1, 3, 4, 5, 6A, 6B, 7F, 9V, 14, 18C, 19F, 19A, 23F) that comprise all serotypes found in lower valency vaccines (PCV7: 4, 6B, 9V, 14, 18C, 19F, 23F; and PCV10: 1, 4, 5, 6B, 7F, 9V, 14, 18C, 19F, 23F), those included in PPV23 (all serotypes included in PCV13 except 6A and serotypes 2, 8, 9N, 10A, 11A, 12F, 15B, 17F, 20, 22F and 33F), and non-vaccine serotypes (NVT). The isolates that were not typable with any of the complete set of sera available from the Staten Serum Institute (Copenhagen, Denmark) were considered non-typable (NT). Given the high frequency of spontaneous switching between serotypes 15B and 15C we opted to include strains with these serotypes into a single group. Due to the difficulty in distinguishing a set of isolates that were positive for both serotypes 25A and 38 we opted to include strains with these serotypes into a single group.

From a total of 1660 isolates recovered, a random sample of ≥50% of the isolates (n = 871) from each serotype and from each year was chosen to be characterized by MLST and tested for the presence of the pilus islands. Briefly, among the 1660 isolates, there were 52 different serotypes, with the 10 most frequent being serotypes 3 (13.0%), 7F (10.0%), 19A (9.8%), 1 (8.5%), 14 (8.1%), 8 (5.7%), 22F (3.9%), 4 (3.2%), 9N (2.8%) and 11A (2.8%). However, the 10 most frequent serotypes were different in each of the age groups. In the 18–49 yr olds (n = 472) these were serotypes 1 (14.0%), 7F (11%), 8 (9.1%), 14 (8.7%), 3 (7.6%), 19A (6.1%), 9N (4.2%), 4 (3.8%), 22F (3.2%), 11A (2.8%). In the 50–64 yr olds (n = 358) these were serotypes 3 (12%), 19A (10.3%), 1 (9.5%), 7F (9.2%), 14 (6.1%), 4 (5.0%), 8 (4.5%), 11A (3.4%), 22F (3.4%), 9V (2.8%). In the ≥65 yr olds (n = 830) these were serotypes 3 (15.8%), 19A (11.6%), 7F (10.2%), 14 (8.7%), 1 (4.9%), 22F (4.6%), 8 (4.2%), 6C (3.0%), 11A (2.5%), 9N (2.3%). Overall, the proportions of PCV7, PCV13 and PPV23 serotypes were 18.4%, 61.9% and 79.4%, respectively. Non-susceptibility to penicillin, defined as either intermediate level penicillin resistance (MIC 0.12–1.0 μg/ml) or high level resistance (MIC≥2.0 μg/ml) as discussed previously [1,12], was found in 330 isolates (19.9%), while 315 isolates (19.0%) were resistant to erythromycin. The age and sex of the patients and the source of the isolates randomly chosen for further study was similar to that of the 1660 isolates. In the genotyped group the age distribution was as follows: 28.5% of the isolates were from individuals 18–49 yrs, 21.2% from 50–64 yrs and 50.3% from ≥65 yrs. The majority of the isolates were collected from blood (87.9%), 8.4% from CSF, 2.5% from pleural fluid and 1.2% from other normally sterile sources.

### MLST

MLST was performed as described previously [13]. The DNA sequences were analyzed using Bionumerics software (Applied-Maths, Sint-Martens-Laten, Belgium) and the alleles and sequence types were assigned according to the pneumococcal MLST database available at http://pubmlst.org/spneumoniae/. The goeBURST algorithm [14] implemented in the PHYLOViZ software [15] was used to establish relationships between STs. Clonal complexes were defined at the single-locus-variant (SLV) and double-locus-variant (DLV) levels.

### Detection of Pilus Islands

The presence of pilus islets (PI) was evaluated by PCR. Briefly, for PI-1 in the absence of the pilus islet, a product of 1-3Kb was expected using primers PFL-up and P-dn flanking the islet [4]. In strains yielding no PCR product, the *rlrA* gene was detected using primers RLRA-up and RLRA-dn. A similar approach was followed to detect the presence of PI-2 [16].

### Statistical Analysis

Sample diversity was evaluated using the Simpson’s index of diversity (SID) and the respective 95% confidence intervals (CI95%) [17]. To compare two sets of partitions the Adjusted Wallace (AW) coefficients were calculated [18] using the online tool available at http://www.comparingpartitions.info. Differences were evaluated by the Fisher exact test with the false discovery rate (FDR) correction for multiple testing [19] and the Cochran-Armitage test was used for trends. A p<0.05 was considered significant for all tests.

## Results

### Sequence Type Distribution and Relationship with Serotype

The 871 isolates analyzed by MLST presented 206 different STs (SID = 0.971, CI95%: 0.967–0.976) grouping into 80 CCs (SID = 0.948, CI95%: 0.942–0.953) according to goeBURST analysis, when including all STs deposited in the database. The 14 most frequent STs, which accounted for more than half of the genotyped isolates (50.6%) were, in decreasing order, ST191 (9.9%), ST306 (7.0%), ST180 (6.9%), ST53 (4.5%), ST156 (4.0%), ST276 (3.6%), ST433 (3.2%), ST66 (2.8%), ST408 (1.7%), ST232 (1.7%), ST260 (1.5%), ST143 (1.4%), ST179 (1.3%) and ST289 (1.3%).

Twenty new allelic combinations and 19 new alleles were identified. The new allelic combinations were identified as STs: 6176, 6177, 6180, 6181, 6182, 6973, 8866, 9955, 9956, 9957, 9958, 9960, 9963, 9966, 9969, 9970, 9971, 9979, 9982 and 9986. The novel alleles identified were designated 200, 307 and 309 for *aroE*, 429, 636 and 637 for *ddl*, 294, 295, 428 and 430 for *gdh*, 437 and 438 for *gki*, 273 for *recP* and 588, 589, 590, 592, 593 and 605 for *xpt*.

There was a strong correlation between CC and the vaccine serotype groups (AW = 0.810, CI95%: 0.763–0.857), with the six most prevalent CCs being mainly composed of isolates presenting vaccine serotypes (95.5%). Table 1 shows the age distribution and serotypes of the most frequent STs found in the 22 major CCs (n≥10 isolates), together accounting for 83.7% of the genotyped isolates. The major CC (CC156, n = 101) included mostly isolates expressing PCV7 serotypes, namely 14, 9V and 23F, while four of the remaining five most frequent CCs were mainly composed of isolates presenting the additional serotypes found in PCV13 (mainly 7F, 3, 1 and 19A). The other most frequent lineage, CC62, consisted mostly of isolates expressing serotypes included only in PPV23 (serotypes 8 and 11A). The age distribution and serotypes of the STs found in CCs with <10 isolates are shown in S1 Table.

**Table 1:** Age distribution and the serotypes of the most frequent STs found in the 22 major CCs (n≥10 isolates) identified by goeBURST.

			no. of isolates per age group				
CC (n)	ST	Total	[18–49]	[50–64]	> = 65	Dominant serotype (n)	Other serotypes
156 (101)	156	35	13	5	17	14 (31)	9V (3), 10A (1)
	143	12	2	2	8	14 (12)	-
	338	10	3	1	6	23F (7)	23A (2), 19F (1)
	162	6	2	1	3	9V (4)	19F (1), 24A (1)
	2944	5	0	3	2	14 (5)	-
	Others^a^	33	8	6	19	9V (10)	14 (8), 6B (5), 6C (3), 23F (3), 35F (2), 17F (1), 17A (1)
191 (88)	191	86	31	15	40	7F (83)	7A (1), NT^b^ (2)
	Others^a^	2	0	2	0	7F (2)	-
180 (68)	180	60	8	17	35	3 (60)	-
	Others^a^	8	2	1	5	3 (8)	-
306 (68)	306	61	26	14	21	1 (61)	-
	350	5	1	2	2	1(5)	-
	Others^a^	2	2	0	0	1(2)	-
62 (67)	53	39	15	8	16	8 (37)	NT^b^ (2)
	408	15	6	4	5	11A (14)	11C (1)
	62	7	1	2	4	11A (7)	-
	Others^a^	6	3	0	3	8 (2), 11A (2)	18C (1), 22F (1)
230 (47)	276	31	3	7	21	19A (31)	-
	230	6	1	1	4	24F (4)	19A (2)
	Others^a^	10	4	3	3	19A (8)	10A (1), 24F (1)
81 (30)	66	24	12	3	9	9N (23)	NT^b^ (1)
	Others^a^	6	1	0	5	24F (4)	4 (2)
433 (29)	433	28	4	5	19	22F (28)	-
	Others^a^	1	0	0	1	22F (1)	-
439 (25)	439	7	0	3	4	23B (7)	-
	42	5	1	3	1	23A (4)	6A (1)
	Others^a^	13	3	2	8	23A (7)	23B (3), 23F (3)
15 (24)	9	8	3	0	5	14 (8)	
	1201	7	3	0	4	19A (4)	7C (3)
	Others^a^	9	1	2	6	14 (5)	34 (2), 6B (1), 7C (1)
177 (24)	179	11	4	4	3	19F (11)	-
	Others^a^	13	3	5	5	19A (5)	19F (3), 21 (3), 15A (1), 15 B/C (1)
199 (21)	416	8	1	2	5	19A (8)	-
	411	7	1	2	4	15B/C (7)	-
	199	6	2	2	2	19A (3)	15B/C (2), 18C (1)
378 (19)	232	15	3	5	7	3 (15)	-
	Others^a^	4	2	0	2	3 (4)	-
113 (16)	123	5	1	0	4	17F (5)	-
	1766	5	2	0	3	31 (5)	-
	Others^a^	6	1	2	3	22F (3)	17F (1), 18C (1), 31 (1)
460 (16)	97	10	2	3	5	10A (10)	-
	Others^a^	6	3	1	2	6A (4)	10A (1), 35F (1)
260 (15)	260	13	2	3	8	3 (13)	-
	Others^a^	2	1	0	1	3 (2)	-
218 (13)	218	10	3	2	5	12B (10)	-
	Others^a^	3	1	1	1	12B (2)	12F (1)
289 (13)	289	11	5	2	4	5 (11)	-
	Others^a^	2	1	0	1	5 (2)	-
30 (11)	30	10	2	2	6	16F (10)	-
	Others^a^	1	0	0	1	16F (1)	-
63 (11)	63	8	3	0	5	15A (7)	15F (1)
	Others^a^	3	0	1	2	3 (1), 7F (1), 15A (1)	-
315 (11)	386	7	1	1	5	6C (6)	6B (1)
	Others^a^	4	0	1	3	6C (3)	6B (1)
404 (10)	404	9	5	0	4	8 (9)	-
	Others^a^	1	1	0	0	8 (1)	-

^a^ Sequence types that accounted for less than 5 isolates each were grouped together in “Others”.

^b^ NT-non typable.

**Fig 1:**
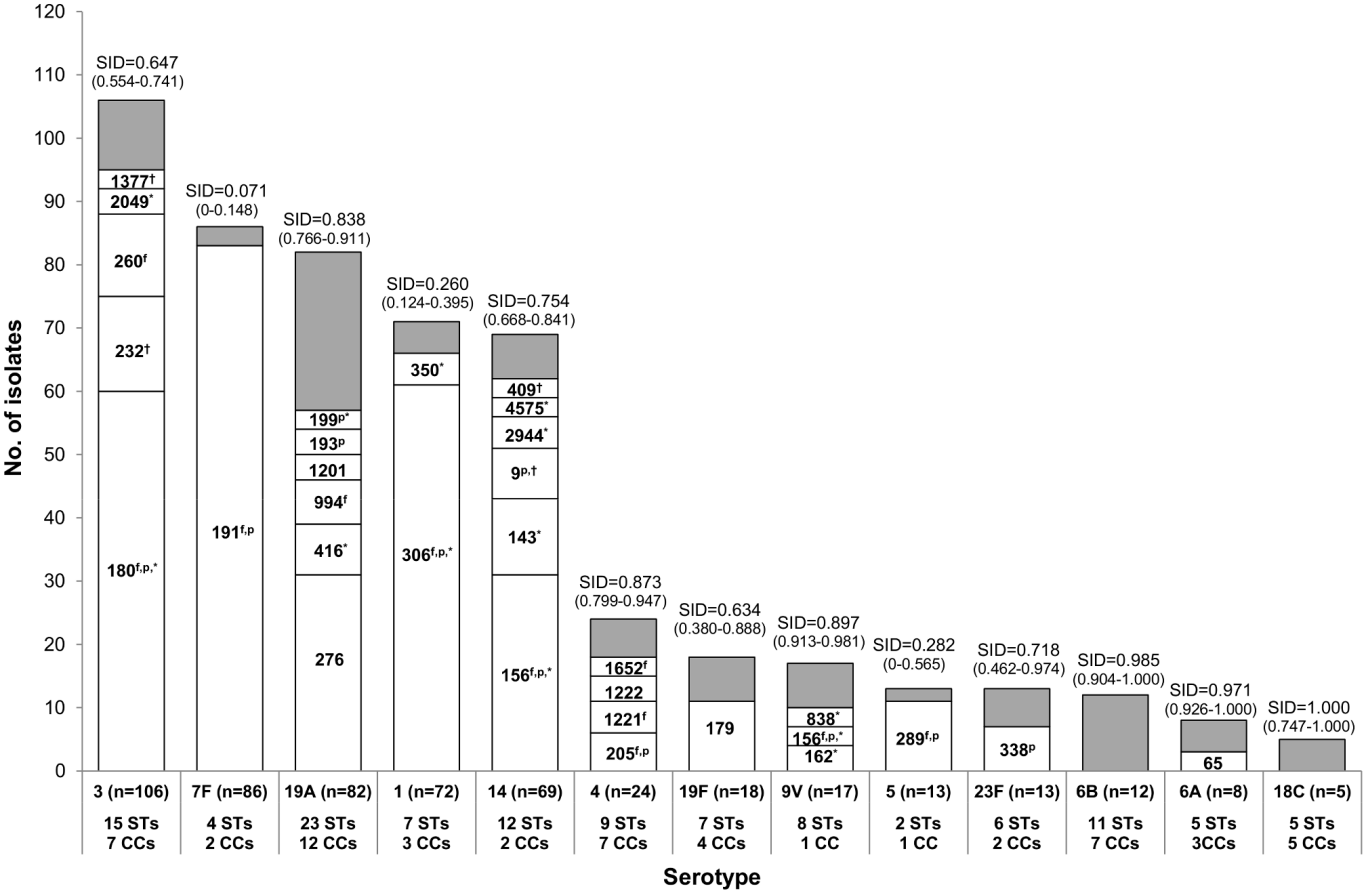
Distribution of STs according to serotype of the isolates causing adult IPD in 2008–2011 and expressing serotypes included in the conjugate vaccines. The STs that were considered by goeBURST as founders of a CC are indicated by “^f^”. The STs that matched the STs of PMEN clones are indicated by “^p^”. Marked either with “*” or “^†^” are STs belonging to the same CC in each serotype. The respective SID values are indicated on top of the bars and in parenthesis are the respective confidence intervals. In grey are represented the isolates included in STs with <3 isolates. These were: **serotype 4** –ST801 (n = 2) and STs 244, 246, 259 and 1866 (n = 1, each); **serotype 6B** –ST176 (n = 2), STs 138, 273, 386, 473, 1518, 6175, 9957, 9970, 9986 and 10051 (n = 1, each); **serotype 9V** –STs 280 and 10044 (n = 2, each) and STs 239, 1762 and 10054 (n = 1, each); **serotype 14** –ST15 (n = 2) and STs 2511, 2616, 4573, 4576 and 10041 (n = 1, each); **serotype 18C** –STs 102, 113, 199, 1233 and 10033 (n = 1, each); **serotype 19F** –ST177 (n = 2), STs 89, 162, 271, 338 and 391 (n = 1, each); **serotype 23F** –ST10039 (n = 2) and STs 1135 and 9579 (n = 1, each); **serotype 1** –STs 217, 228, 1233, 3081 and 4578 (n = 1, each); **serotype 3** –ST1220 (n = 2) and STs 505, 1230, 6014, 9162 and 10038 (n = 1, each); **serotype 5** –STs 280 and 10044 (n = 2, each), STs 239, 1762 and 10054 (n = 1, each); **serotype 6A** –ST1876 (n = 2) and STs 42, 460 and 10055 (n = 1, each); **serotype 7F** –STs 1062, 1589 and 3130 (n = 1, each) and **serotype 19A** –STs 230, 242, 320, 2013 and 6174 (n = 2, each) and STs 241, 878, 2102, 2669, 2732, 4197, 4847, 6178, 6973, 9963 and 10042 (n = 1, each).

**Fig 2:**
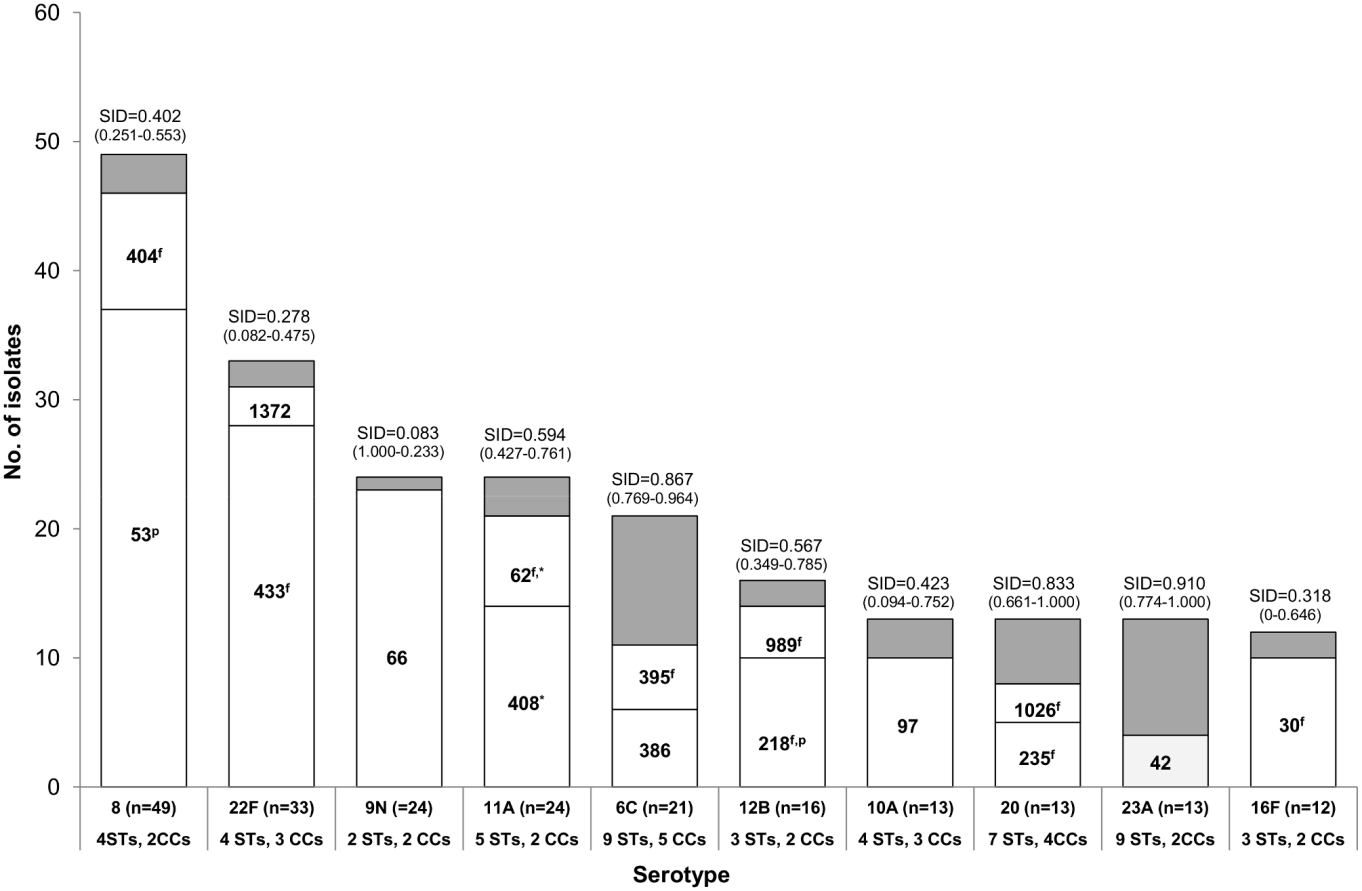
Distribution of STs according to serotype of the isolates causing adult IPD in 2008–2011 and expressing the 10 most frequent serotypes not included in any of the conjugate vaccines. The respective SID values are shown on top of the bars and in parenthesis are the respective confidence intervals. In grey are represented the isolates included in STs with <3 isolates. These were: **serotype 8** –ST1012 (n = 2) and ST 9969 (n = 1); **serotype 22F** –STs 10053 and 10220 (n = 1, each); **serotype 9N** –ST3982 (n = 1); **serotype 11A** –STs 9955, 9960 and 10052 (n = 1, each); **serotype 6C** –STs 1150, 1692 and 3396 (n = 2, each) and STs 1390, 1715, 2667 and 4310 (n = 1, each); **serotype 12B** –ST6180 (n = 2); **serotype 10A** –STs 156, 816 and 3135 (n = 1, each); **serotype 20** –STs 1483, 1871, 7221, 9958 and 10047 (n = 1, each); **serotype 23A** –ST338 (n = 2) and STs 190, 311, 438, 6177, 7960, 8866 and 10048 (n = 1, each); **serotype 16F** –STs 570 and 5902 (n = 1, each).

Fig 1 shows the STs expressing each of the 13 serotypes included in PCV13 and Fig 2 the STs expressing each of the 10 most frequent serotypes found among those not included in any of the conjugate vaccines. The STs found in the remaining serotypes are indicated in S2 Table. The genetic diversity varied greatly with serotype, with serotypes, 4, 6A, 6B, 9V, 18C, 19A, 20 and 23A being highly diverse (SID>0.8) and serotypes 1, 5, 7F, 9N and 22F displaying very limited diversity (SID<0.3). In general, there was a predominance of high genetic diversity among PCV13 serotypes and low genetic diversity among the 10 most frequent non-PCV13 serotypes. For serotypes 9V, 14 and 23A, the wide variety of STs did not result in a high diversity of CCs, with a maximum of two CCs being detected in each. The genetic diversity of each serotype was independent of the serotype’s frequency. Examples of this are the low frequency serotypes 6B and 18C that presented a high genetic diversity and no dominant ST.

A total of 587 isolates (67.3%) presented STs related to 29 of the 43 clones recognized by the Pneumococcal Molecular Epidemiology Network (PMEN) [20], sharing at least five MLST alleles with these clones (357 isolates had the same ST, 133 were SLVs and 97 were DLVs). When considering these isolates the predominant clones were Netherlands^7F^-191 (n = 88), Spain^9V^-156 (n = 71), Netherlands^3^-180 (n = 68), Netherlands^8^-53 (n = 63), Sweden^1^-306 (n = 63), Denmark^14^-230 (n = 47), Tennessee^14^-67 (n = 24), Tennessee^23F^-37 (n = 24) and Netherlands^15B^-199 (n = 21) (Figs 1 and 2 and S2 Table). Additionally, another 63 isolates were included in the same CCs of other four PMEN clones.

The correlation between ST and serotype was high (AW = 0.942, CI95%: 0.912–0.973), but there were STs that presented more than one serotype (Table 1 and S1 and S2 Tables). The serotype distribution along the studied years for the STs expressing more than one serotype is shown in Table 2.

**Table 2:** Serotype distribution for the STs expressing more than one serotype between 2008–2011.

	Serotype (n)			
ST^a^ (n)	2008	2009	2010	2011
156 (35)	14 (6), 9V (1)	14 (12), 9V (1)	14 (7), 10A (1)	14 (6), 9V (1)
338 (10)	-	23F (3)	23F (2), 23A (1), 19F (1)	23F (2), 23A (1)
717 (9)	-	33A (1)	33A (4), 33F (1)	33A (2), 3 (1)
63 (8)	15A (2)	15A (3)	15A (2), 15F (1)	-
386 (7)	-	6C (2)	6C (2)	6C (2), 6B (1)
1201 (7)	19A (2), 7C (1)	19A (1), 7C (1)	19A (1), 7C (1)	-
162 (6)	9V (3), 19F (1)	-	9V (1)	24A (1)
199 (6)	15B/C (1)	15B/C (1), 19A (2), 18C (1)	-	19A (1)
230 (6)	24F (2)	-	24F (2), 19A (1)	19A (1)
42 (5)	6A (1)	23A (2)	23A (2)	-
241 (5)	18A (3)	-	18A (1), 19A (1)	-

^a^ Only the sequence types that presented ≥ 5 isolates are shown.

### Variation of STs with Time

When analyzing the evolution of STs between 2008 and 2011 we identified some fluctuations, although the majority reflected changes in serotype prevalence occurring in this period. However, while for ST306 (serotype 1) there was a decline, significant after correcting for multiple testing (from 11.0% to 2.8%, Cochran-Armitage test of trend p = 0.014), for the other STs the changes were only significant before FDR correction. The STs for which there was a significant p-value in the Cochran-Armitage test for trends but unsupported after FDR correction were: ST53 (serotype 8), that increased from 3.3% to 6.5% (p = 0.043); ST289 (serotype 5), that accounted for 2.4% of IPD in 2008 and 0% in 2011 (p = 0.020); ST717 (serotypes 33A and 33F) that increased from 0% to 1.4% (p = 0.048); and STs 193 (serotype 19A) and 409 (serotype 14) that were only detected in 2008 (1.9% and 1.4%, respectively; p = 0.001 and p = 0.020, respectively). Regarding changes in CCs with time, these reflected the changes identified in STs, with only CC306 declining significantly after FDR correction (from 12.9% to 2.8%, Cochran-Armitage test of trend p = 0.001).

### Relationship of STs with Patient Age and Isolate Source

When grouping the isolates according to the three patient age groups– 18–49 yrs, 50–64 yrs and > = 65 yrs—only CC5902 showed a statistically significant association with age. The seven isolates belonging to this CC were all recovered from individuals with 18–49 yrs (p = 0.011, significant after FDR correction, S1 Table).

When testing for associations between STs and CCs and isolate source, the only significant association found was between CC460 and CSF, with 6 out of 16 isolates being collected from CSF (p = 0.012, significant after FDR correction).

### Presence of Pilus Islands

A total of 278 isolates, representing 31.9% of the genotyped collection, carried at least one PI. Among these, 107 (38.5%) had only PI-1, 165 (59.4%) only PI-2 and 6 (2.2%) presented the two PIs simultaneously.

While the proportion of PI-1 positive isolates remained stable between 2008 and 2011 (from 10.0% to 11.6%, Cochran-Armitage test of trend p = 0.857), there was a significant decline of PI-2 carrying isolates (from 24.8% to 15.8%, Cochran-Armitage test of trend p = 0.007). This also resulted in an overall increase in the proportion of isolates lacking any of the pilus islands, from 63.8% in 2008 to 72.6% in 2011 (p = 0.013).

The presence and variants of the PIs were more strongly associated with ST (AW = 0.950, CI95%: 0.933–0.967) than with serotype (AW = 0.711, CI95%: 0.651–0.771). The STs that were significantly associated with PI-1 and PI-2 are shown in Table 3. All isolates included in CC320 (n = 3) and CC2669 (n = 3) presented the two PIs simultaneously.

**Table 3:** Sequence types that were associated with pilus island 1 (PI-1) and pilus island 2 (PI-2).

Type of Pilus	ST	Yes	No	OR^a^ (95% CI)	P-value^b^
Pilus 1	156	26	9	24.68 (10.79–61.89)	<0.001
	143	12	0	Inf (20.32-Inf)	<0.001
	179	10	1	72.89 (10.18–3133.46)	<0.001
	416	8	0	Inf (12.04-Inf)	<0.001
	162	6	0	Inf (8.16-Inf)	<0.001
	205	6	0	Inf (8.16-Inf)	<0.001
	2944	5	0	Inf (6.30-Inf)	<0.001
	1221	5	0	Inf (6.30-Inf)	<0.001
	4575	3	0	Inf (2.8-Inf)	0.002
	838	3	0	Inf (2.8-Inf)	0.002
	191	0	86	0 (0–0.27)	<0.001
	306	0	61	0 (0–0.39)	<0.001
	180	0	60	0 (0–0.40)	<0.001
Pilus 2	191	86	0	Inf (181.20-Inf)	<0.001
	306	61	0	Inf (99.04-Inf)	<0.001
	350	5	0	Inf (3.81-Inf)	<0.001
	180	0	60	0 (0–0.24)	<0.001
	53	0	39	0 (0–0.39)	<0.001
	156	0	35	0 (0–0.44)	<0.001

^a^ OR—Odds ratio.

^b^ Only significant values after FDR correction are shown.

Among the 105 isolates presenting only PI-1, 87.9% expressed PCV7 serotypes, namely serotypes 14 (n = 49), 4 (n = 15), 19F (n = 13), 9V (n = 11) and 6B (n = 6). The remaining isolates were from serotypes 19A (n = 9) and 7F, 24A and 35B (n = 1, each). PI-2 positive isolates were from serotypes 7F (n = 85), 1 (n = 68), 11A (5), 19A (n = 2), 3, 7A and 31 (n = 1, each) and NT (n = 2). The isolates presenting simultaneously the two types of PIs were from serotypes 19A (n = 5) and 19F (n = 1).

No associations between isolate source and type of PI were detected. Still, there was a low proportion of PI-2 positive isolates among isolates recovered from the CSF, with only 6 of the 73 CSF isolates presenting PI-2, and while 7 of the 15 isolates recovered from pleural fluid carried PI-2, none carried PI-1.

### Antimicrobial Resistance

Similarly to pilus islands, resistance to antimicrobials was more strongly associated with ST than with serotype. The AW for ST or serotype and penicillin susceptibility was, respectively, 0.785 (CI95%: 0.729–0.841) and 0.389 (CI95%: 0.326–0.452), while the AW for ST or serotype and erythromycin susceptibility was, respectively, 0.711 (CI95%: 0.598–0.824) and 0.315 (CI95%:0.217–0.413). The sequence types that were associated with penicillin non-susceptible pneumococci (PNSP) and erythromycin resistant pneumococci (ERP) are presented in Table 4.

**Table 4:** Sequence types that were positively associated with penicillin non-susceptibility, erythromycin resistance, erythromycin and penicillin non-susceptibility simultaneously and multi drug resistance.

Antimicrobial resistance^a^	ST	Yes	No	OR^b^ (95% CI)	P-value^c^	Penicillin MIC range (μg/ml)
PNSP	156	34	1	153.83 (25.29–6036.47)	<0.001	0.5–3
	276	31	0	Inf (34.58-Inf)	<0.001	0.19–3
	143	12	0	Inf (10.84-Inf)	<0.001	0.75–3
	338	10	0	Inf (8.65-Inf)	<0.001	0.064–0.19
	63	8	0	Inf (6.52-Inf)	<0.001	0.094–1
	386	7	0	Inf (5.48-Inf)	<0.001	0.064–0.19
	179	7	4	6.69 (1.68–31.50)	<0.001	0.047–2
	230	6	0	Inf (4.45-Inf)	<0.001	0.38–0.75
	2944	5	0	Inf (3.45-Inf)	<0.001	2–8
ERP	276	30	1	150.50 (24.56–5946.55)	<0.001	0.19–3
	179	11	0	Inf (10.96-Inf)	<0.001	0.047–2
	143	10	2	21.90 (4.60–206.95)	<0.001	0.75–3
	717	9	0	Inf (8.52-Inf)	<0.001	0.008–0.032
	9	8	0	Inf (7.32-Inf)	<0.001	0.016–0.064
	63	8	0	Inf (7.32-Inf)	<0.001	0.094–1
	386	7	0	Inf (6.15-Inf)	<0.001	0.064–0.19
	350	5	0	Inf (3.87-Inf)	<0.001	0.004–0.023
	230	5	1	21.27 (2.36–1006.44)	0.001	0.38–0.75
EPNSP	276	30	1	274.20 (44.39–10466.80)	<0.001	0.19–3
	143	10	2	36.81 (7.69–350.14)	<0.001	0.75–3
	63	8	0	Inf (12.17-Inf)	<0.001	0.064–0.19
	386	7	0	Inf (10.19-Inf)	<0.001	0.064–0.19
	179	7	4	12.52 (3.12–59.31)	<0.001	0.047–2
	230	5	1	35.15 (3.88–1669.53)	<0.001	0.38–0.75
	4575	3	0	Inf (2.83-Inf)	0.002	2–3

^a^ PNSP—Penicillin non-susceptible pneumococci, ERP—Erythromycin resistant pneumococci, EPNSP—Erythromycin and penicillin non-susceptible pneumococci.

^b^ OR—odds ratio. Inf—infinite.

^c^ Only significant values after FDR correction are shown.

## Discussion

In spite of several years of PCV7 use in children, the most frequent CC was CC156 (11.6%, Table 1), a lineage that expressed mainly PCV7 serotypes (89.1%) and which was also the most frequent in IPD in the pre-PCV7 period [11]. We had previously shown that the serotype distribution of pneumococci causing adult IPD had changed significantly in the post-PCV7 period, with the proportion of PCV7 serotypes declining to values below 20% [1,12,21]. Adult vaccination with anti-pneumococcal vaccines was low to negligible and prior work indicated that these changes were due to a combination of secular trends and herd effect from children vaccination, which although occurring through the private market reached a coverage of 75% of children ≤2 yrs in 2008 [1,12,21]. Due to these changes one could expect that CC156 would also decrease (this CC accounted for 21.7% of all IPD in 1999–2003 [11]) and potentially lose its dominance. During the study period CC156 accounted for an approximately constant proportion of the characterized isolates in each year (varying slightly between 5.5% and 7.0%). The observed persistence of this CC may be explained by three different factors: 1) while it is true that PCV7 serotypes have declined in importance, it is also true that they still account for approximately one fifth of adult IPD and 57% of the isolates expressing PCV7 serotypes in 2008–2011 belonged to this CC; 2) this CC is strongly associated with antimicrobial resistance, with n = 70/101 isolates being resistant to at least two different classes of antibiotics; and 3) the genomic diversity of CC156 is high, with one study reporting the presence of 10 unrelated genetic subgroups [22], suggesting that this CC may be particularly suited to adapt to different selective pressures. Regarding the last point, in our study we found representatives of three different clones recognized by the PMEN included in CC156: Spain^9V^-156, Colombia^23F^-338 and Greece^6B^-273 [20].

Overall, the clones recognized by the PMEN were strongly represented in our collection with up to 67.3% of the isolates being at most DLVs of one of the 29 different PMEN clones identified. Among the 22 major CCs occurring in the study period (Table 1), only six did not include a PMEN clone: CC433, CC378, CC460, CC260, CC30 and CC404. The most frequent of these, CC433 (mainly ST433, Table 1), was the eighth most frequent CC, included mostly isolates susceptible to antimicrobials, and is now an important cause of IPD worldwide [23–27].

The eight more frequent CCs (Table 1) were mainly composed of isolates expressing one of the top 10 serotypes causing adult IPD in 2008–2011, excluding serotype 4 that presented a high genetic diversity and no dominant CC (Fig 1). In fact, the clonal composition of the 10 most frequent serotypes causing adult IPD in Portugal in 2008–2011 (Figs 1 and 2) presented both similarities and differences with other geographic regions in similar periods, with most matching results coming from countries in Europe and the Americas, especially for serotypes 3 (Netherlands^3^-180), 7F (Netherlands^7F^-191), 22F (ST433) and 9N (ST66) [23–26,28–30]. Most of these lineages, with the exception of ST66, were also dominant among isolates expressing the same serotypes and causing IPD in children in Japan [27]. Among the isolates expressing serotypes 19F and 23F, the lineages that dominated in the present study where either absent or represented a minority of the isolates of the same serotype in the recent studies from the United States and Japan [26,27], indicating the persistence of different lineages expressing PCV7 serotypes in different countries. Serotype 19A, which increased as a cause of IPD after PCV7 implementation in several countries, was associated in Portugal with the expansion of the PMEN clone Denmark^14^-230 while in the USA and Asia it was associated with the emergence of the PMEN clone Taiwan^19F^-236, as previously described [3]. Serotype 1 was mostly represented by the Sweden^1^-306 European clone [31]. However, we detected for the first time in Portugal two serotype 1 isolates belonging to the hypervirulent PMEN clone Sweden^1^-217 (STs 217 and 3081), which has been responsible for epidemics with high mortality in Africa [31,32]. The detection of these genotypes in Portugal is not surprising, since they were found in neighboring Spain [28] and Portugal has a significant community of citizens of African descent. Still, the two isolates detected were collected in 2011, the last year of the study period, so it will be important to monitor the potential emergence of this genotype as a cause of adult IPD in Portugal. Serotypes 14 and 8 were found mainly among representatives of Spain^9V^-156 and Netherlands^8^-53, respectively, similarly to Spain [28]. Serotype 11A was found mainly among representatives of ST408 in our study, while the most common lineage in both Spain and the USA was its SLV, ST62 [26,28]. For serotype 4, in spite of the higher diversity some similarity was also found with Spain, with Sweden^4^-205 and ST246 being common to the two collections of isolates [28].

When comparing our results with those from a recent carriage study in adults in Portugal [33] in addition to the difference in serotype distribution due to the recognized differences in invasiveness of the various serotypes [2], there was also a marked difference between the clonal compositions of serotype 19A, since the majority of isolates expressing this serotype among asymptomatic carriers represented ST1201 (CC15), while in our study the most frequent was ST276, indicating possible differences in virulence between these two serotype 19A lineages.

After the introduction of PCV7, several studies documented a general decrease in IPD incidence. However, the benefits of vaccination were also partly overcome by increases in incidence of non-vaccine serotypes [5,7,34,35]. This could occur through the persistence of a successful lineage now expressing a different serotype not covered by the conjugate vaccines, a phenomenon described as capsular switching. Among our collection a notable case of possible capsular switching was the detection of five isolates related to the PMEN clone Denmark^14^-230 (ST230, n = 4 and ST4253, n = 1) expressing the non-PCV13 serotype 24F (Table 2). This combination has already been reported in Portugal in colonized children [36], in Italy [37], Spain and other European countries (http://pubmlst.org/). In Portugal, in the pre-PCV7 period, serotype 24F was predominantly CC81 and mostly susceptible to antimicrobials. In 2008–2011, among the nine isolates genotyped, four represented CC81 and were mainly antimicrobial susceptible as before, while five represented CC230 and were EPNSP. The detection of this genotype expressing serotype 24F in Portugal is of concern since ST276, an SLV of ST230, was behind the expansion of serotype 19A as a cause of IPD in Portugal in the post-PCV7 era [3]. Among other possible capsular switches detected in our collection (Table 2), most reflected the occasional detection of a single isolate of a different serotype, suggesting that even if these result from capsular switching they did not persist in the population at a significant frequency. Taken together this data indicates that capsular switching in our collection was infrequent and cannot be attributed to vaccine pressure, in agreement with other studies [38,39]. However, even though these events were rare they can be important since the uncommon combinations may proliferate in the future if the conditions become favorable maintaining successful clones in circulation.

Clonal expansion of previously less frequent lineages was a major contributor to the expansion of non-PCV7 serotypes, since the 22 most frequent CCs occurring in 2008–2011 (Table 1) were already in circulation in 1999–2003 [11]. When comparing these two periods the most relevant changes were the expansion of CC191 (serotype 7F) and CC439 (serotypes 23B and 23A) and the decline of CC260 and CC458 (both associated with serotype 3), CC1381 (serotype 18C) and that of CC156 discussed above. The variations in frequency of CC191, CC439 and CC1381 followed the changes occurring in the respective serotypes. Regarding the clonal composition of serotype 3, we found that the decrease in CC260 and CC458 was accompanied by an expansion of CC180 among serotype 3 isolates, explaining the relative stability of this serotype among IPD in adults [12], with CC180 accounting for 40% of serotype 3 IPD in 1999–2003 but for 64% in 2008–2011. Given that isolates belonging to CC180, CC260 or CC458 were mostly susceptible to all tested antimicrobials and that only one isolate from CC180 and another from CC458 carried a PI, this different behavior in time cannot be attributed to differences in these characteristics.

The presence and type of the PIs was more strongly associated with genotype than with serotype, as previously reported [4]. The genotypes that carried PIs in our study (Table 3) were essentially the same reported recently in USA [26], although the proportions of these genotypes differed considerably between the two studies. The proportion of PI-1 carrying isolates increased in the post-PCV7 period in the USA associated with the emergence of the non-PCV7 serotypes 19A and 35B [40]. Although serotype 19A also increased in Portugal, the genotype behind this increase does not carry a PI (ST276) and an actual decrease of PI-1 positive isolates occurred when compared to the pre-PCV7 period, when 24% of the adult isolates presented PI-1 [4]. The proportion of isolates presenting only PI-2 declined during the study period, from 25% in 2008 to 15% in 2011. This was expected since serotype 1 isolates are significantly associated with PI-2 and these decreased as a cause of adult IPD during the study period [12]. Since PCV13 also includes serotype 7F, which in Portugal was strongly associated with PI-2, continued use of PCV13 may further reduce the proportion of isolates carrying PI-2. In 2011, the proportion of isolates carrying any of the PIs was down to 26.6% of the isolates. As suggested for isolates causing IPD in children [16], continued PCV13 use has the potential to virtually eliminate PI carrying isolates.

Antimicrobial resistance is not a crucial pre-requisite for the success of serotypes in IPD, as demonstrated by serotypes 1, 3 and 7F that were frequent in the post-PCV7 period and are mostly susceptible to antimicrobials. Still, the presence of resistant clones may help the persistence of serotypes targeted by vaccines, as was possibly the case with serotypes 14 and 19A. The highest proportions of penicillin and erythromycin resistance among adult IPD since the beginning of epidemiological surveillance were registered in 2010, although these declined again in 2011 [12]. Between 2008 and 2009, when only the increase in PNSP was significant, this was due to an increase in PNSP expressing serotypes 14 and 19A. In contrast, between 2009 and 2010, the increase in both PNSP and ERP was due to an increase in genetically unrelated resistant isolates expressing different serotypes. Since the number of isolates collected yearly between 2008 and 2011 did not suffer significant fluctuations, two possibilities could explain the initial increase in PNSP isolates expressing serotypes 14 and 19A: 1) an increase in the overall proportions of serotypes 14 and 19A, including PNSP STs or 2) an increase in the proportion of PNSP STs within each of these serotypes, with a concomitant decrease of susceptible STs. Regarding serotype 14 isolates, which increased slightly during the study period, these were by 2008 almost equally distributed into only two CCs: CC15, which includes ST409 and that is almost entirely penicillin susceptible, and CC156, in which all serotype 14 isolates were PNSP. From 2009 onwards, CC156 became the dominant lineage, accounting for over 90% of the isolates expressing serotype 14, a change that was not only due to a decline in frequency of CC15 but also to a slight overall increase in frequency of CC156 among all adult IPD isolates. Among serotype 19A isolates, the increase in proportion of PNSP between 2008 and 2009 was due to the disappearance of ST193, which was fully susceptible to penicillin, and to an increase of ST276, which represented solely PNSP isolates (Table 4). Although PNSP and ERP returned in 2011 to values similar to those found prior to 2010, this was due to a decrease in frequency of resistant isolates representing multiple STs and expressing different serotypes, while the emerging clones (CC156 among serotype 14 and ST276 among serotype 19A) persisted as important causes of adult IPD. Continued surveillance of resistant isolates should focus particularly on the evolution of serotype 24F since ≥50% of the isolates expressing this serotype in our study were associated with the PMEN clone Denmark^14^-230 (S2 Table) which was a major clone in the expansion of serotype 19A in the post-PCV7 period in Portugal.

The significant differences in genetic variation, as documented here by MLST, within the various serotypes remain unexplained and should be the object of future study. We have shown that the changes in serotypes occurring during the study period have been driven mostly by the expansion of previously circulating clones or to declines in the majority of the lineages expressing a given serotype. However, in some serotypes, such as 14 and 19A, changes in serotype frequency were driven mostly by changes in particular lineages. In the case of serotype 3, although its proportion remained constant with time, there were significant changes in the dominant lineages. These observations raise the possibility that lineage-specific properties may condition the dynamics of particular serotypes. Serotype switching played a minor role in this population but may be an important source of new variants that may increase in the post PCVs period. Taken together, these observations reinforce the importance of determining the clonal lineages of pneumococci to better understand the changes in the bacterial population occurring following the use of PCVs.

## Supporting Information

S1 Table Age distribution and serotypes of the STs found in CCs with less than 10 isolates. (PDF)

S2 Table Distribution of STs according to serotype of the isolates (n≤11) causing adult IPD in 2008–2011 and expressing serotypes not included in any of the conjugate vaccines. (PDF)

## References

[1] Horácio AN, Diamantino-Miranda J, Aguiar SI, Ramirez M, Melo-Cristino J, the Portuguese Group for the Study of Streptococcal Infections. Serotype changes in adult invasive pneumococcal infections in Portugal did not reduce the high fraction of potentially vaccine preventable infections. Vaccine. 2012;30: 218–224. 10.1016/j.vaccine.2011.11.022 22100892

[2] Sá-Leão R, Pinto F, Aguiar S, Nunes S, Carriço JA, Frazão N, et al Analysis of invasiveness of pneumococcal serotypes and clones circulating in Portugal before widespread use of conjugate vaccines reveals heterogeneous behavior of clones expressing the same serotype. J Clin Microbiol. 2011;49: 1369–1375. 10.1128/JCM.01763-10 21270219 PMC3122870

[3] Aguiar SI, Pinto FR, Nunes S, Serrano I, Melo-Cristino J, Sá-Leão R, et al Denmark^14^-230 clone as an increasing cause of pneumococcal infection in Portugal within a background of diverse serotype 19A lineages. J Clin Microbiol. 2010;48: 101–108. 10.1128/JCM.00665-09 19864476 PMC2812288

[4] Aguiar SI, Serrano I, Pinto FR, Melo-Cristino J, Ramirez M. The presence of the pilus locus is a clonal property among pneumococcal invasive isolates. BMC Microbiol. 2008;8: 41 10.1186/1471-2180-8-41 18307767 PMC2270847

[5] Aguiar SI, Brito MJ, Gonçalo-Marques J, Melo-Cristino J, Ramirez M. Serotypes 1, 7F and 19A became the leading causes of pediatric invasive pneumococcal infections in Portugal after 7 years of heptavalent conjugate vaccine use. Vaccine. 2010;28: 5167–5173. 10.1016/j.vaccine.2010.06.008 20558247

[6] Bettinger JA, Scheifele DW, Kellner JD, Halperin SA, Vaudry W, Law B, et al The effect of routine vaccination on invasive pneumococcal infections in Canadian children, Immunization Monitoring Program, Active 2000–2007. Vaccine. 2010;28: 2130–2136. 10.1016/j.vaccine.2009.12.026 20044050

[7] Pilishvili T, Lexau C, Farley MM, Hadler J, Harrison LH, Bennett NM, et al Sustained reductions in invasive pneumococcal disease in the era of conjugate vaccine. J Infect Dis. 2010;201: 32–41. 10.1086/648593 19947881

[8] Rodenburg GD, de Greeff SC, Jansen AGCS, de Melker HE, Schouls LM, Hak E, et al Effects of pneumococcal conjugate vaccine 2 years after its introduction, the Netherlands. Emerg Infect Dis. 2010;16: 816–823. 10.3201/eid1605.091223 20409372 PMC2953990

[9] Beall B, McEllistrem MC, Gertz RE, Wedel S, Boxrud DJ, Gonzalez AL, et al Pre- and postvaccination clonal compositions of invasive pneumococcal serotypes for isolates collected in the United States in 1999, 2001, and 2002. J Clin Microbiol. 2006;44: 999–1017. 16517889 10.1128/JCM.44.3.999-1017.2006 PMC1393141

[10] Pai R, Moore MR, Pilishvili T, Gertz RE, Whitney CG, Beall B. Postvaccine genetic structure of *Streptococcus pneumoniae* serotype 19A from children in the United States. J Infect Dis. 2005;192: 1988–95. 16267772 10.1086/498043

[11] Serrano I, Melo-Cristino J, Carriço JA, Ramirez M. Characterization of the genetic lineages responsible for pneumococcal invasive disease in Portugal. J Clin Microbiol. 2005;43: 1706–1715. 10.1128/JCM.43.4.1706-1715.2005 15814989 PMC1081348

[12] Horácio AN, Diamantino-Miranda J, Aguiar SI, Ramirez M, Melo-Cristino J, the Portuguese Group for the Study of Streptococcal Infections. The majority of adult pneumococcal invasive infections in Portugal are still potentially vaccine preventable in spite of significant declines of serotypes 1 and 5. PLoS ONE. 2013;8: e73704 10.1371/journal.pone.0073704 24066064 PMC3774749

[13] Enright MC, Spratt BG. A multilocus sequence typing scheme for *Streptococcus pneumoniae*: identification of clones associated with serious invasive disease. Microbiology. 1998;144: 3049–60. 9846740 10.1099/00221287-144-11-3049

[14] Francisco AP, Bugalho M, Ramirez M, Carriço JA. Global optimal eBURST analysis of multilocus typing data using a graphic matroid approach. BMC Bioinformatics. 2009;10: 152 10.1186/1471-2105-10-152 19450271 PMC2705362

[15] Francisco AP, Vaz C, Monteiro PT, Melo-Cristino J, Ramirez M, Carriço JA. PHYLOViZ: Phylogenetic inference and data visualization for sequence based typing methods. BMC Bioinformatics. 2012;13: 87 10.1186/1471-2105-13-87 22568821 PMC3403920

[16] Aguiar SI, Melo-Cristino J, Ramirez M. Use of the 13-valent conjugate vaccine has the potential to eliminate pilus carrying isolates as causes of invasive pneumococcal disease. Vaccine. 2012;30: 5487–5490. 10.1016/j.vaccine.2012.06.062 22749798

[17] Carriço JA, Silva-Costa C, Melo-Cristino J, Pinto FR, de Lencastre H, Almeida JS, et al Illustration of a common framework for relating multiple typing methods by application to macrolide-resistant *Streptococcus pyogenes*. J Clin Microbiol. 2006;44: 2524–32. 16825375 10.1128/JCM.02536-05 PMC1489512

[18] Severiano A, Pinto FR, Ramirez M, Carriço JA. Adjusted Wallace coefficient as a measure of congruence between typing methods. J Clin Microbiol. 2011;49: 3997–4000. 10.1128/JCM.00624-11 21918028 PMC3209087

[19] Benjamini Y, Hochberg Y. Controlling the false discovery rate—a practical and powerful approch to multiple testing. J R Stat Soc Ser B Stat Methodol. 1995;57: 289–300.

[20] McGee L, McDougal L, Zhou J, Spratt BG, Tenover FC, George R, et al Nomenclature of major antimicrobial-resistant clones of *Streptococcus pneumoniae* defined by the pneumococcal molecular epidemiology network. J Clin Microbiol. 2001;39: 2565–71. 11427569 10.1128/JCM.39.7.2565-2571.2001 PMC88185

[21] Aguiar SI, Serrano I, Pinto FR, Melo-Cristino J, Ramirez M. Changes in *Streptococcus pneumoniae* serotypes causing invasive disease with non-universal vaccination coverage of the seven-valent conjugate vaccine. Clin Microbiol Infect. 2008;14: 835–843. 10.1111/j.1469-0691.2008.02031.x 18844684

[22] Moschioni M, Lo Sapio M, Crisafulli G, Torricelli G, Guidotti S, Muzzi A, et al Sequence analysis of 96 genomic regions identifies distinct evolutionary lineages within CC156, the largest Streptococcus pneumoniae clonal complex in the MLST database. PloS One. 2013;8: e61003 10.1371/journal.pone.0061003 23593373 PMC3625235

[23] Pichon B, Ladhani SN, Slack MPE, Segonds-Pichon A, Andrews NJ, Waight PA, et al Changes in Molecular Epidemiology of Streptococcus pneumoniae Causing Meningitis following Introduction of Pneumococcal Conjugate Vaccination in England and Wales. J Clin Microbiol. 2013;51: 820–827. 10.1128/JCM.01917-12 23269742 PMC3592052

[24] Ardanuy C, Tubau F, Pallares R, Calatayud L, Dominguez MA, Rolo D, et al Epidemiology of invasive pneumococcal disease among adult patients in Barcelona before and after pediatric 7-valent pneumococcal conjugate vaccine introduction, 1997–2007. Clin Infect Dis. 2009;48: 57–64. 10.1086/594125 19035779

[25] Golden AR, Adam HJ, Gilmour MW, Baxter MR, Martin I, Nichol KA, et al Assessment of multidrug resistance, clonality and virulence in non-PCV-13 *Streptococcus pneumoniae* serotypes in Canada, 2011–13. J Antimicrob Chemother. 2015;70: 1960–1964. 10.1093/jac/dkv061 25761605

[26] Metcalf BJ, Gertz RE Jr, Gladstone RA, Walker H, Sherwood LK, Jackson D, et al Strain features and distributions in pneumococci from children with invasive disease before and after 13-valent conjugate vaccine implementation in the USA. Clin Microbiol Infect. 2016;22: 60.e9–60.e29. 10.1016/j.cmi.2015.08.027 PMC4721534 26363404

[27] Nakano S, Fujisawa T, Ito Y, Chang B, Suga S, Noguchi T, et al Serotypes, antimicrobial susceptibility, and molecular epidemiology of invasive and non-invasive Streptococcus pneumoniae isolates in paediatric patients after the introduction of 13-valent conjugate vaccine in a nationwide surveillance study conducted in Japan in 2012–2014. Vaccine. 2016;34: 67–76. 10.1016/j.vaccine.2015.11.015 26602268

[28] Muñoz-Almagro C, Ciruela P, Esteva C, Marco F, Navarro M, Bartolome R, et al Serotypes and clones causing invasive pneumococcal disease before the use of new conjugate vaccines in Catalonia, Spain. J Infect. 2011;63: 151–162. 10.1016/j.jinf.2011.06.002 21679725

[29] Yildirim I, Stevenson A, Hsu KK, Pelton SI. Evolving picture of invasive pneumococcal disease in massachusetts children: a comparison of disease in 2007–2009 with earlier periods. Pediatr Infect Dis J. 2012;31: 1016–1021. 10.1097/INF.0b013e3182615615 22673142

[30] Caierão J, Hawkins P, Sant’anna FH, da Cunha GR, d’Azevedo PA, McGee L, et al Serotypes and genotypes of invasive Streptococcus pneumoniae before and after PCV10 implementation in southern Brazil. PloS One. 2014;9: e111129 10.1371/journal.pone.0111129 25356595 PMC4214725

[31] Brueggemann AB, Spratt BG. Geographic distribution and clonal diversity of *Streptococcus pneumoniae* serotype 1 isolates. J Clin Microbiol. 2003;41: 4966–4970. 14605125 10.1128/JCM.41.11.4966-4970.2003 PMC262517

[32] Harvey RM, Stroeher UH, Ogunniyi AD, Smith-Vaughan HC, Leach AJ, Paton JC. A variable region within the genome of *Streptococcus pneumoniae* contributes to strain-strain variation in virulence. PloS One. 2011;6: e19650 10.1371/journal.pone.0019650 21573186 PMC3088708

[33] Almeida ST, Nunes S, Santos Paulo AC, Valadares I, Martins S, Breia F, et al Low prevalence of pneumococcal carriage and high serotype and genotype diversity among adults over 60 years of age living in Portugal. PloS One. 2014;9: e90974 10.1371/journal.pone.0090974 24604030 PMC3946249

[34] Aguiar SI, Brito M, Horácio AN, Lopes J, Ramirez M, Melo-Cristino J, et al Decreasing incidence and changes in serotype distribution of invasive pneumococcal disease in persons aged under 18 years since introduction of 10-valent and 13-valent conjugate vaccines in Portugal, July 2008 to June 2012. Euro Surveill. 2014;19: pii: 20750. 10.2807/1560-7917.es2014.19.12.20750 24698140

[35] Waight PA, Andrews NJ, Ladhani SN, Sheppard CL, Slack MPE, Miller E. Effect of the 13-valent pneumococcal conjugate vaccine on invasive pneumococcal disease in England and Wales 4 years after its introduction: an observational cohort study. Lancet Infect Dis. 2015;15: 535–543. 10.1016/S1473-3099(15)70044-7 25801458

[36] Simões AS, Pereira L, Nunes S, Brito-Avô A, de Lencastre H, Sá-Leão R. Clonal evolution leading to maintenance of antibiotic resistance rates among colonizing pneumococci in the PCV7 era in Portugal. J Clin Microbiol. 2011;49: 2810–2817. 10.1128/JCM.00517-11 21632898 PMC3147772

[37] Pantosti A, Gherardi G, Conte M, Faella F, Dicuonzo G, Beall B. A novel, multiple drug-resistant, serotype 24F strain of *Streptococcus pneumoniae* that caused meningitis in patients in Naples, Italy. Clin Infect Dis Off Publ Infect Dis Soc Am. 2002;35: 205–208. 10.1086/341250 12087529

[38] Wyres KL, Lambertsen LM, Croucher NJ, McGee L, von Gottberg A, Liñares J, et al Pneumococcal capsular switching: an historical perspective. J Infect Dis. 2013;207: 439–49. 10.1093/infdis/jis703 23175765 PMC3537446

[39] Ramirez M, Tomasz A. Acquisition of new capsular genes among clinical isolates of antibiotic-resistant *Streptococcus pneumoniae*. Microb Drug Resist. 1999;5: 241–246. 10647080 10.1089/mdr.1999.5.241

[40] Regev-Yochay G, Hanage WP, Trzcinski K, Rifas-Shiman SL, Lee G, Bessolo A, et al Re-emergence of the type 1 pilus among Streptococcus pneumoniae isolates in Massachusetts, USA. Vaccine. 2010; 10.1016/j.vaccine.2010.04.042 PMC2897942 20434550

