# Supplementary material for: Population Structure of Streptococcus pneumoniae Causing Invasive Disease in Adults in Portugal before PCV13 Availability for Adults: 2008-2011
Source: PLoS One. 2016 May 11;11(5):e0153602. doi: 10.1371/journal.pone.0153602 (PMC4864403; doi:10.1371/journal.pone.0153602)
Supplement: S1 Table — (PDF) [file pone.0153602.s001.pdf]

Table S1: Age distribution and serotypes of the STs found in CCs with less than 10 isolates.

CC (no.isolates)	ST (no.isolates)	no. of isolates per age group			Serotypes (no.isolates)
		[18-49]	[50-64]	>=65	
717 (9)	717 (9)	0	3	6	33A (7), 33F (1), 3 (1)
994/6158 (9)	994 (7)	1	1	5	19A (7)
	4197 (2)	0	0	2	19A (2)
235 (8)	235 (5)	2	1	2	20 (5)
	1483 (1)	0	1	0	20 (1)
	7221 (1)	0	0	1	20 (1)
	10047 (1)	0	0	1	20 (1)
395 (7)	395 (5)	2	1	2	6C (5)
	1692 (2)	0	0	2	6C (2)
205 (6)	205 (6)	4	0	2	4 (6)
5902 (6)	1222 (4)	4	0	0	4 (4)
	801 (2)	2	0	0	4 (2)
	5902 (1)	1	0	0	16F (1)
241 (5)	241 (5)	2	0	3	18A (4), 19A (1)
473 (5)	1876 (2)	0	1	1	6A (2)
	473 (1)	0	0	1	6B (1)
	1135 (1)	0	0	1	23B (1)
	10055 (1)	1	0	0	6A (1)
1221 (5)	1221 (5)	2	2	1	4 (5)
393 (4)	393 (4)	0	0	4	25A (4)
989 (4)	989 (4)	1	2	1	12B (4)
1816 (4)	1342 (3)	1	0	2	29 (3)
	2567 (1)	0	0	1	29 (1)
320 (3)	320 (2)	0	1	1	19A (2)
	271 (1)	0	0	1	19F (1)
1026 (3)	1026 (3)	1	1	1	20 (3)
1046 (3)	1046 (2)	1	0	1	34 (2)
	8967 (1)	0	0	1	34 (1)
1652/6956 (3)	1652 (3)	0	3	0	4 (3)
2669 (3)	2669 (2)	0	1	1	19A (2)
	2102 (1)	0	0	1	19A (1)
2690 (3)	2690 (3)	0	0	3	35B (3)
198 (2)	198 (1)	0	0	1	35B (1)
	3329 (1)	1	0	0	35B (1)
217 (2)	217 (1)	1	0	0	1 (1)
	3081 (1)	0	1	0	1 (1)
242 (2)	242 (2)	0	0	2	19A (2)
452 (2)	452 (1)	0	1	0	35B (1)
	9979 (1)	0	1	0	29 (1)
546 (2)	494 (2)	0	1	1	28A (2)

CC (no.isolates)	ST (no.isolates)	no. of isolates per age group			Serotypes (no.isolates)
		[18-49]	[50-64]	>=65	
1381 (2)	1233 (2)	2	0	0	1 (1), 18C (1)
2021 (2)	1116 (1)	1	0	0	3 (1)
	1126 (1)	0	1	0	39 (1)
2599 (2)	2599 (2)	1	0	1	17A (2)
9958 (2)	9958 (2)	1	0	1	18A (1), 20 (1)
1368 (2)	1368 (2)	0	1	1	35F (2)
100 (2)	100 (2)	0	0	2	33A (1), 33F (1)
70 (1)	70 (1)	1	0	0	13 (1)
87 (1)	89 (1)	0	1	0	19F (1)
102 (1)	102 (1)	1	0	0	18C (1)
259 (1)	259 (1)	0	1	0	4 (1)
343 (1)	10049 (1)	0	0	1	15A (1)
432 (1)	432 (1)	1	0	0	21 (1)
458 (1)	458 (1)	0	1	0	3 (1)
558 (1)	558 (1)	0	1	0	35B (1)
901 (1)	901 (1)	0	1	0	13 (1)
1010 (1)	9955 (1)	0	0	1	11A (1)
1025 (1)	1025 (1)	0	0	1	15B/C (1)
1151 (1)	2732 (1)	0	0	1	19A (1)
1390 (1)	1390 (1)	0	0	1	6C (1)
1866 (1)	1866 (1)	0	0	1	4 (1)
1884 (1)	478 (1)	1	0	0	NT (1)
2658 (1)	2658 (1)	0	0	1	13 (1)
3214 (1)	3214 (1)	0	0	1	35F (1)
3982 (1)	3982 (1)	0	0	1	9N (1)
6182 (1)	6182 (1)	0	0	1	36 (1)
7069 (1)	7069 (1)	0	0	1	15A (1)
8153 (1)	5823 (1)	0	1	0	28F (1)
9957 (1)	9957 (1)	0	1	0	6B (1)
9970 (1)	9970 (1)	0	1	0	6B (1)
10043 (1)	10043 (1)	1	0	0	29 (1)
10051 (1)	10051 (1)	0	0	1	6B (1)
1083/7843 (1)	1083 (1)	1	0	0	25F (1)
1715/1640 (1)	1715 (1)	1	0	0	6C (1)
6973/2668 (1)	6973 (1)	0	1	0	19A (1)
