# Supplementary material for: Population Structure of Streptococcus pneumoniae Causing Invasive Disease in Adults in Portugal before PCV13 Availability for Adults: 2008-2011
Source: PLoS One. 2016 May 11;11(5):e0153602. doi: 10.1371/journal.pone.0153602 (PMC4864403; doi:10.1371/journal.pone.0153602)
Supplement: S2 Table — (PDF) [file pone.0153602.s002.pdf]

Table S2. Distribution of STs according to serotype of the isolates (n≤11) causing adult IPD in 2008-2011 and expressing serotypes not included in any of the conjugate vaccines.

Serotype (no. Isolates)	ST (no. Isolates)
15A (11)	63 (7)
	1228 (1)
	3130 (1)
	7069 (1)
	10049 (1)
15B/C (11)	199 (2)
	411 (7)
	1025 (1)
	3863 (1)
23B (11)	439 (7)
	1135 (1)
	9579 (1)
	10039 (2)
24F (9)	72 (4)
	230 (4)
	4253 (1)
29 (8)	198 (1)
	558 (1)
	1342 (3)
	2567 (1)
	9979 (1)
	10043 (1)
33A (8)	100 (1)
	717 (7)
17F (7)	123 (5)
	6179 (1)
	9966 (1)
31 (6)	1766 (5)
	1994 (1)
35F (6)	1366 (2)
	1368 (2)
	3214 (1)
	4849 (1)
Non-typable (6)	53 (2)
	66 (1)
	191 (2)
	478 (1)
34 (5)	1046 (2)
	2001 (2)
	8967 (1)

Serotype (no. Isolates)	ST (no. Isolates)
35B (5)	452 (1)
	2690 (3)
	3329 (1)
18A (5)	241 (4)
	9958 (1)
18C (5)	102 (1)
	113 (1)
	199 (1)
	1233 (1)
	10033 (1)
7C (4)	1201 (3)
	9956 (1)
25A (4)	393 (4)
21 (4)	432 (1)
	1877 (3)
17A (3)	392 (1)
	2599 (2)
13 (3)	70 (1)
	901 (1)
	2658 (1)
28A (2)	494 (2)
33F (2)	100 (1)
	717 (1)
15F (1)	63 (1)
36 (1)	6182 (1)
11C (1)	408 (1)
25F (1)	1083 (1)
24A (1)	162 (1)
28F (1)	5823 (1)
39 (1)	1126 (1)
12F (1)	220 (1)
7A (1)	191 (1)
